# Healthy Design and Urban Planning Strategies, Actions, and Policy to Achieve Salutogenic Cities

**DOI:** 10.3390/ijerph15122698

**Published:** 2018-11-29

**Authors:** Stefano Capolongo, Andrea Rebecchi, Marco Dettori, Letizia Appolloni, Antonio Azara, Maddalena Buffoli, Lorenzo Capasso, Alessandra Casuccio, Gea Oliveri Conti, Alessandro D’Amico, Margherita Ferrante, Umberto Moscato, Ilaria Oberti, Lorenzo Paglione, Vincenzo Restivo, Daniela D’Alessandro

**Affiliations:** 1 Dipartimento di Architettura, Ingegneria delle Costruzioni e Ambiente Costruito, Politecnico di Milano, 20133 Milan, Italy; stefano.capolongo@polimi.it (S.C.); maddalena.buffoli@polimi.it (M.B.); ilaria.oberti@polimi.it (I.O.); 2 Dipartimento di Scienze Mediche, Chirurgiche e Sperimentali, Università degli Studi di Sassari, 07100 Sassari, Italy; azara@uniss.it; 3 Dipartimento di Ingegneria Civile Edile e Ambientale, Sapienza Università di Roma, 00184 Rome, Italy; letizia.appolloni@uniroma1.it (L.A.); alessandro.damico@uniroma1.it (A.D.); lorenzo.paglione@uniroma1.it (L.P.); daniela.dalessandro@uniroma1.it (D.D.); 4 Dipartimento di Sanità Pubblica, Medicina Sperimentale e Forense, Università degli Studi di Pavia, 27100 Pavia, Italy; lorenzo.capasso@unipv.it; 5 Dipartimento di Scienze per la Promozione della Salute e Materno Infantile, Università degli Studi di Palermo, 90133 Palermo, Italy; alessandra.casuccio@unipa.it (A.C.); vincenzo.restivo@unipa.it (V.R.); 6 Dipartimento di Scienze Mediche Chirurgiche e Tecnologie Avanzate, Università degli Studi di Catania, 95131 Catania, Italy; olivericonti@unict.it (G.O.C.); marfer@unict.it (M.F.); 7 Fondazione Policlinico Agostino Gemelli IRCCS, Università Cattolica del Sacro Cuore, 00168 Rome, Italy; umberto.moscato@unicatt.it

**Keywords:** urban health, public health, salutogenic cities, healthy urban planning strategies, health promotion, non-communicable disease prevention

## Abstract

Starting from a previous experience carried out by the working group “Building and Environmental Hygiene” of the Italian Society of Hygiene and Preventive Medicine (SItI), the aim of the present work is to define new strategic goals for achieving a “Healthy and Salutogenic City”, which will be useful to designers, local governments and public bodies, policy makers, and all professionals working at local health agencies. Ten key points have been formulated: 1. climate change and management of adverse weather events; 2. land consumption, sprawl, and shrinking cities; 3. tactical urbanism and urban resilience; 4. urban comfort, safety, and security perception; 5. strengths and weaknesses of urban green areas and infrastructures; 6. urban solid waste management; 7. housing emergencies in relation to socio-economic and environmental changes; 8. energy aspects and environmental planning at an urban scale; 9. socio-assistance and welfare network at an urban scale: importance of a rational and widespread system; and 10. new forms of living, conscious of coparticipation models and aware of sharing quality objectives. Design strategies, actions, and policies, identified to improve public health and wellbeing, underline that the connection between morphological and functional features of urban context and public health is crucial for contemporary cities and modern societies.

## 1. Introduction

In the contemporary international scenario, the World Health Organization (WHO) recognizes the influence adequate indoor and outdoor living conditions have on public health, with particular attention paid to densely built contexts [1]. Domestic accidents, indoor air quality, water supply, management and collection of municipal solid waste, and green and blue areas represent features of the built environment that could directly and indirectly impact on citizens’ health, without neglecting the key role of the living environments in protecting living conditions against climate changes [2]. Adequate living conditions thus necessitate healthy environments and promoters of active lifestyles; individual and collective health is strongly influenced by the environmental context [3].

The phenomenon of urbanization, taking place on a global scale, mostly regards the densely built urban environment as a living space for a growing number of individuals, with estimates of 60% of the world’s population destined to live in medium–large cities by 2050 [4].

At present, the urban environment and modern cities are pivotal for the “public health paradox”: on the one hand, they should respond to urbanization by offering shelter and protection to the population, promoting and protecting collective health; on the other hand, urban areas require high energy consumption, contextually representing a source of multiple risk factors for health issues. For example, the urban heat island effect and urban pollution are two major interconnected problems of the urban environment that have become more serious with rapid urbanization and both the removal and loss of urban green spaces [5], and affect human health, causing an increase in cases of cardiovascular and pulmonary morbidity and mortality. Although the global surface occupied by urban areas is only 2%, cities produce the largest share of greenhouse gasses and deplete natural resources to meet the growing demand for goods by individuals [6].

The resulting impact is the price that the same urban areas pay: natural and non-natural disasters, extreme weather phenomena, and catastrophes increasingly hit cities, causing devastating environmental, economic, and social implications. According to the Center for Epidemiology of Disaster [7], in 2017, there were 335 disasters worldwide that caused 9607 victims and affected 96 million people, amounting in 334 billion dollars of damage caused.

Nowadays, cities must face the resulting challenge of climatic migrations of an increasing number of individuals. Recently, the World Bank declared that internal climate migrants are rapidly becoming the human face of climate change. By 2050, climate change could force more than 143 million individuals to leave their homes as a result of adverse weather phenomena [8]. This exodus will mainly be directed towards urban areas and cities, where migrants hope to find refuge from poverty, but which often have hidden risks of social and health inequalities.

Individual and collective health is linked to the general and local policies that establish the strict dependence between the morphological and functional organization of the urban context and public health [9], a crucial aspect that, while offering clear opportunities for health protection and promotion, also poses underlying risks. In particular, globalization and urbanization, together with the aging of the population, interact with the determinants of social, cultural, and economic health (education, living, and working spaces), exposing people to behavioral risk factors (smoking habits, unhealthy food, reduced physical activity, alcohol abuse, etc.), which can cause the emergence of chronic and degenerative diseases [10,11].

According to WHO, modern cities must deal with three major health problems: infectious and acute diseases, non-communicable diseases, and accidents linked to the safety of cycle–pedestrian accessibility and mobility [12]. In order to face this challenge, a proper planning and organization of the urban environment is pivotal in Italy [13], as well as in Europe and worldwide, and it is thus crucial to consider cities as "enhancers" of indoor [14] and outdoor [15] living conditions [16,17], as well as public health.

## 2. Background

Taking all of this into account, this research work, proposed by the working group “Building and Environmental Hygiene” of the Italian Society of Hygiene and Preventive Medicine (SItI), represents an update of the most important risk factors and related “Healthy Urban Planning” actions, strategies, and policies for the improvement of public health and wellbeing in contemporary cities.

A previous, similar, and important experience, carried out by the same SItI working group, was the 50th course of the “International School of Epidemiology and Preventive Medicine G D’Alessandro” entitled “Urban Health. Instruments for promoting health and for assessing the hygienic and sanitary conditions in urban areas”. It was an occasion for both professors and attendees to highlight the key role of urban planning as a fundamental support for healthy and sustainable lifestyles and for reducing inequalities in health conditions [18]. At the end of the scientific debate, the Erice 50 Charter [19], entitled “Strategies for Diseases Prevention and Health Promotion in Urban Areas”, which includes the key points shown in Table 1, was published.

The ten previously mentioned key points represent the outcome of an experience of scientific debate held during a residential course with professors, students, and experts on the subject, to highlight the emerging urban and public health problems of relevant importance at the time of the workshop. The working group “Building and Environmental Hygiene” of SItI has set itself the goal of continuing to investigate the middleic of urban health during its annual activities, through studies and research funded by the Italian Ministry of Health, as well as scientific dissemination in various Italian institutions and universities. At present, the authors, who are part of this group, starting with an update of the literature, consider it useful to publish an incremental renewal of the key strategic objectives, in line with the emerging environmental, public, and urban health issues, with particular reference to the Italian context and the national scenario.

## 3. Purpose

According to the above hypotheses, this document intends to integrate the previously defined strategic objectives, focusing above all on possible responses to the emerging environmental, social, and economic criticalities that have characterized recent years. It aims to direct territorial planning not only towards “Healthy cities”, but also towards “Salutogenic cities”. In our view, the contents of this document may provide a useful basis for designers, local governments and public bodies, policy makers, and all professionals working at local health agencies.

## 4. The Key Strategic Objectives

Ten new key points have been formulated, integrated with the previous ones.

The work of integration has focused its attention on three main specific objectives: (1) environmental and social sustainability of urban areas; (2) urban environment adaptation to climatic changes; and (3) new responses to the population’s housing needs. These three objectives are further divided into ten sub-objectives (key points), as indicated in Table 2.

### 4.1. Climate Change and Management of Adverse Weather Events

Several scientists have clarified that global warming and climate change are unequivocal facts [20]. Epidemiologists have highlighted how climate change affects human health, reporting some alarming results. Today, extreme weather such as heat-waves, cold spells, floods, dust storms, and hurricanes are the major climatic issues that need to be managed or mitigated, as these events are more frequent than in the past. Extreme weather affects human morbidity and mortality, especially in regard to urban citizens [21,22,23].

Populations are directly and indirectly exposed to climate change. These exposures can result in a variety of negative health impacts [24,25,26], including epigenetic effects, for example, because of exposure to smoke [27,28] and deaths, which can potentially be prevented through early warning systems, as well as through both forecasts and a timely response by public health bodies [29].

The findings of a recent Canadian study [30] highlight that a concerted effort of Public Health managers, together with electrical and fuel providers, in response to floods, dust storms, and hurricanes can reduce some vulnerabilities that could impact access to food, especially for urban populations. Interruptions to electricity supply due to extreme weather conditions, for example, could have negative impacts on food access and food safety, opening the way to infectious diseases in humans.

Practical, feasible, repeatable strategies and actions that could be adopted are listed below:

➢ to provide updated guidelines and strategies about “heat-health action plans”, referring to the experience acquired in different countries [20];
➢ to supply better link alerts to effective communication and intervention strategies to reduce heat-related mortality;
➢ to improve mitigation tools against the impact of soil dusts through the introduction or preservation of green plants and woods;
➢ to promote actions to mitigate these risks that must include addressing food access vulnerabilities through ongoing city-wide strategies and integrating food access into the city’s emergency response planning with a management of the “last mile” of food distribution with the development of food resilience action plans for vulnerable areas [30];
➢ to tackle the root causes of climate change, understand the public health co-benefits of several actions with a valorization of healthy environments, and advocate health-related developments is vital in order to reduce the burden of disease and promote population health.

### 4.2. Land Consumption, Sprawl, and Shrinking Cities

Urban sprawl is an unplanned incremental urban development, characterized by a low-density mix of land uses on the urban fringe, and is often associated with shrinkage when the cities undergo population losses and economic transformation for more than two years.

Worldwide demographic development has some variations among countries and several indices have been tested to measure urban sprawl, showing an increasing trend of urban sprawl in the USA and Europe in recent decades, although research on this middleic is still in progress [31,32,33].

The sprawling nature of cities is critical because of its major impacts on increased energy, and land and soil consumption. These impacts threaten both the natural and rural environments, raising greenhouse gas emissions that cause climate change, and elevated air and noise pollution levels, which often exceed the agreed human safety limits. Thus, urban sprawl produces many adverse impacts that have direct effects on the quality of life for people living in cities.

Practical, feasible, repeatable strategies and actions that could be adopted are listed below:

➢ to underline that effective policymaking requires a reliable understanding of the state of the environment and the present rates of environmental change. The objectives of environmental monitoring are to document environmental conditions and to detect and better understand changes;
➢ to elaborate on the high sensitivity and specificity sprawl index that can greatly help land-use planners to critically assess projected plans, and control urban sprawl and its negative consequences. Measuring only the extent of built-up areas is not sufficient to reveal the full extent of these patterns;
➢ to consider a sprawl index that combines several socio-economic variables, that is, those related to transport infrastructure (road density and railway density), demography (population density and age structure), the economic situation (gross domestic product and employment ratio), governmental effectiveness, and changes in lifestyle (e.g., household size and car ownership), which are influential drivers of urban sprawl.
➢ to validate a tailored sprawl index that has to be coupled with emission reduction strategies including promotion of active transportation (i.e., vehicles, fuels, vehicle miles traveled) to realize exposure reductions across the entire population;
➢ to implement a scheduled monitoring of urban sprawl in European environmental legislation, which will be useful to design health-promoting public green spaces in the urban environment that have the potential to be health-promoting in multiple dimensions (e.g., increasing physical activity, decreasing exposure to air pollution, and improving mental health).

### 4.3. Tactical Urbanism and Urban Resilience

“Urban resilience” is defined as “the ability of a system, community, or society exposed to hazards to resist, absorb, accommodate, and recover from the effects of a hazard in a timely and efficient manner, including through the preservation and restoration of its essential basic structures and functions” [34]. Building urban resilience requires moving from a tactical approach, focused on the immediate reaction to the event, to a strategic one, focused on long-term management [35]. In order to reach the aim, it is important to look at a city holistically, in order to understand the systems that make up the city and the potential risks it may face. These stresses could be chronic (such as those derived from climate changes, endemic violence, and poverty) or shocking (such as natural or man-made disasters). This is crucial to the understanding of how to allow the city to improve its development and protect the well-being of its citizens.

At the same level, the term “tactical urbanism” describes a set of low-cost, temporary, and scalable changes to the built environment to catalyze long-term interventions. Indeed, it is an approach of actions and policies aimed at improving local neighborhoods and city gathering places. Tactical urbanism initiatives represent a phenomenon that has been spreading in recent years, as an alternative and a challenge to formal spatial planning tools to meet the need for a more responsive planning system [36]. In other words, it is a process of social legitimation of the transformations aimed at providing a real and immediate response to the inhabitants’ demands and spatial needs. Direct impacts on public health are mainly represented by the reduction of social inequalities and the improvement of healthy behaviors [37].

Practical, feasible, repeatable strategies and actions that could be adopted are listed below:

➢ to move from a tactical approach, focused on the immediate reaction to the event, to a strategic one, focused on long-term management;
➢ to improve disaster preparation to ensure an effective first response, and to implement “build back better” practices in recovery, rehabilitation, and reconstruction [38];
➢ to improve resilience of health infrastructure, cultural heritage, and work-places [38];
➢ to remove imminent health risks caused by poor living conditions and inaccessibility to basic services. It allows residents to think beyond basic vulnerabilities and start developing coping strategies for potential disturbances [39];
➢ to promote social initiatives aimed at triggering and accompanying a real innovation process of conventional policies;
➢ to raise awareness among policy makers in generating a differentiation between plan making and plan implementation;
➢ to encourage training opportunities for designers, aimed at acquiring the skills to deal with the existing tension between the need to involve the inhabitants in the processes of a rethinking of the spaces and the need to obtain solutions with formal quality.

### 4.4. Urban Comfort, Safety, and Security Perception

Fear of crime is one of the most significant social problems in cities [40]. In general, this type of urban insecurity is related to other uncertainties regarding labor, economic, or emotional concerns, as well as social insecurities arising from changes in welfare state policies. In general, the most severe expression has been found in badly maintained housing estates with large housing blocks, little maintenance, and large public open spaces with unclear management responsibilities [41]. Some factors appear to reduce the fear of crime at a neighborhood scale; namely, street lighting improvements, multi-component environmental crime prevention programs, or regeneration programs [42], although more research is needed on individual components and effects in various business settings [43].

Practical, feasible, repeatable strategies and actions that could be adopted are listed below:

➢ to conduct environmental audits of public spaces that are commonly identified as unsafe, followed by community generated programs to improve the appearance of neighborhoods [44];
➢ to design neighborhood strategies to enhance community cohesion;
➢ to promote community justice initiatives to improve responsibility for behavior in offenders and enhance victim satisfaction with the justice system;
➢ to discourage behavior that triggers fear utilizing ‘alcohol-free zones’ and other strategies;
➢ to take into consideration strategies that encourage women to report violence and harassment and to access support services;
➢ to increase public awareness of any reduction in crime rates or in risk factors associated with fear of crime;
➢ to increase the role of police as purveyors of reassurance toward the messages of fear of politics and the media;
➢ to design lots, streets, and houses to encourage interaction between neighbors.

### 4.5. Strengths and Weaknesses of Urban Green Areas and Infrastructures

Green areas play an important role in improving the environmental quality and climate of the city [45], although several aspects related to health impacts have not been fully investigated to date. In particular, the presence of green areas has many benefits for the environment and community, such as air quality; social cohesion; and mental health, with particular reference to stress and physical activity levels [46]. A point on which it is considered useful to dwell is, instead, that related to potential health risks (especially in the most vulnerable subjects) connected above all to the exposure to the allergenic pollen of plants (especially Anemophilous) and to the emission of biogenic volatile organic compounds (BVOC) [47]. Moreover, it has been demonstrated that a change in the climatic conditions modifies the growth area of plants that find new growth opportunities even in areas that were previously climatically prohibitive [48].

Practical, feasible, repeatable strategies and actions that could be adopted are listed below:

➢ to design and implement proper maintenance of green areas;
➢ to choose, in the green areas projects, the essences that must take into account the possible impact on the population and habitat (e.g., plants with greater capacity to absorb CO_2_ [49], avoiding species that release known allergens and allergic parasites and species that produce large quantities of volatile isoprenoids);
➢ to increase the safety perception of the green spaces both in terms of use and equipment and social security (e.g., through proper artificial lighting and integration of services);
➢ to develop strategies that ensure accessibility to all users, both for reachability (e.g., proximity of green areas, visibility of accesses, etc.) and usability (e.g., availability of routes, physical usability of pedestrian routes in relation to the different types of user, etc.);
➢ to utilize equipment and street furniture in conformity with the requisites of usability, pleasure, and quality over time;
➢ to consider strategies that encourage the involvement of the local community in the design of green areas.

### 4.6. Urban Solid Waste Management

Starting from the Agenda 21, in which one of the last categories refers to “environmentally sound management of solid wastes and sewage-related issues”, and Agenda 2030, in which the Sustainable Development Goal (UN, 2015) number 11 discusses “sustainable cities and communities”, the common objective is to understand how the urban solid waste management practices and policies affect public health issues. Another recent and important document is the “23 Global Cities and Regions Advance Towards Zero Waste” (C40 CITIES program), which states that the commitment will avoid disposal of at least 87 million tons of waste by 2030 [50].

Traditionally, municipalities have focused only on the environmental issues. Nowadays, the provision of urban solid waste services, including waste collection, transfer, recycling, resource recovery, and disposal, is well developed by several European and international cities like Milan, Oslo, Stockholm, and Vancouver, which are trying to innovate the collection systems located in the urban context, providing greater efficiency and effectiveness, as well as security and prevention from potential infectious risks. These problems and issues are more relevant in the metropolis of developing countries, where the urbanization phenomenon could become an uncontrolled source of risks for public health [51]—the reason why much scientific research has been conducted [52]. For these purposes, it is important to identify exportable and repeatable best practices, starting from the assumption that urban solid waste management should become an integral part of urban planning and architectural design processes.

Practical, feasible, repeatable strategies and actions that could be adopted are listed below:

➢ to involve the population in collective and attentive ways that guarantee a high degree of supervision over the correctness of the differentiated waste collection;
➢ to program innovative digital and smart devices, such as a smartphone application, capable of facilitating differentiated waste collection and making citizens active participants in the processes of reporting anomalous situations, thus obtaining a capillary control over the whole city. Moreover, the complete automation of sorting operations, through an optical reader installed in the collection stations, saves time and increases the efficiency of the system;
➢ to disseminate "returnable" practice as in Milan and Stockholm, which allow an immediate and significant reduction of waste;
➢ to replace regular bins with smart bins, which could allow a careful and continuous monitoring of waste collection, especially in public spaces, which are neglected on numerous occasions.
➢ to increase pneumatic networks like the “Automated Vacuum Waste Collection System” (Oslo and Stockholm), which replace garbage trucks (pollutant emissions and road congestion would be reduced) and the regular garbage collections, ensuring a safe and protected provision to the specialized centers. The limited size necessary for the waste disposal columns allows for their integration with the urban furniture, respecting the aesthetic perception of the places.

### 4.7. Housing Emergencies in Relation to Socio-Economic and Environmental Changes

Italy, as well as Europe and several countries worldwide, is currently undergoing a housing crisis, and this is leading to the residential use of inadequate spaces, or spaces not normally considered as living environments [53]. Italy has a very fragile territory, and climate changes have been adding serious problems in recent years, in particular, floods, landslides, and earthquakes have scourged the national territory with serious repercussions on the quality of the urban and indoor environment [53,54].

Practical, feasible, repeatable strategies and actions that could be adopted are listed below:

In order to minimize the impact of housing emergencies, various proposals have been developed by national and international groups [55,56,57,58,59], which could be summarized in the following key points:

➢ to update the building regulations, and to coordinate them with the sanitary ones in general, and in particular with those regarding housing;
➢ to find a clear and univocal expression for the hygienic requirements of buildings at the local level, surpassing the dualism between municipal building regulations and local hygiene regulations, and referring to a territory wider than the municipal one;
➢ when selecting sites for temporary housing, it is essential to consider safe water supply; sufficient surface area; autonomous thermo-energy systems; natural drainage; psycho-social, economic, physical, and political security [60]; access to communication routes; safe food supply; possibility of returning to their homes; ownership of the land; future enlargement possibility;
➢ minimum hygienic requirements for the population must be the following: proper disposal of waste and wastewater; safe water supply [61,62,63,64]; protection against the vectors of infectious diseases; avoiding overcrowding; ensuring a correct indoor micro-climate; limiting exposure to fine and coarse powders, fungal spores, toxic and radioactive materials, and mineral fibers [65,66]; minimizing the psycho-social aspects of relocation;
➢ only temporarily can the recommendations of the UN Refugee Agency (UNCHR) in terms of building planning requirements be applied. These are as follows: area/person (30 sq.m.); covered space/person (3.5 sq.m.); no. of people/water point (250); no. of people/latrines (20); distance from the water point (150 m at most); distance from water point to latrines (30 m); point to light fire (75 every 300 m); distance between two houses (2 m at least).

### 4.8. Energy Aspects and Environmental Planning at Urban Scale

To satisfy all the parameters of indoor (heating, cooling, or lighting) and outdoor well-being (urban comfort), it is often necessary to use high amounts of energy. There are, however, passive strategies and devices (which do not require the use of energy) capable of limiting energy consumption and, at the same time, ensuring high standards of comfort. Their use limits the demand for energy and, consequently, the impacts of its production, thus protecting health and the environment. A recent literature review highlighted that several non-communicable diseases, like cardio-respiratory ones and psychological stress, are strictly related to factors such as overcrowding, the local heat island effect, and air pollution coming from traffic and domestic heating systems [67].

Practical, feasible, repeatable strategies and actions that could be adopted are listed below:

➢ to minimize the buildings’ energy demand, starting from a correct study of the roads and block orientation regarding the sun and dominant winds;
➢ to maximize the efficiency of energy conversion technologies;
➢ to optimize the water cycle by correctly monitoring a closed cycle (collection, sedimentation, storage, and delivery systems) and promoting sustainable urban drainage systems (green roofs, porous pavements, water squares, rain gardens, etc.);
➢ to control the thermal properties of urban surfaces, with particular reference to heat storage and solar radiation reflection;
➢ to guarantee evapotranspiration through green areas;
➢ to properly design the urban air flows and wind paths (urban canyon geometry and wind penetration in the built area) using breezeway, low-rise buildings and linear parks. In the European climate, it will be important to limit urban winter ventilation and to encourage summer ventilation.

### 4.9. Socio-Assistance and Welfare Network at an Urban Scale: Importance of a Rational and Widespread System

Programming in the widespread availability and accessibility of the social and health services’ network requires a growing integration between institutions and disciplines [68]. This concerns the geography of the city, the planning of public transport and communication routes [69], the urban planning of the neighborhoods, and the usability of public and private spaces. Finally, it requires an extended view of public health, through the “equity lens” [70].

Factors that influence the accessibility of services are in fact both “spatial” and “aspatial” [71]. Spatial factors require a systematic approach in the planning of the surrounding spaces, starting from an assessment of the potential catchment area and its movements. Aspatial factors involve questioning the linguistic, cultural, and social barriers that can render the service ineffective.

All of these different approaches are the basis of the “medical neighborhood” concept, which represents the way in which several national healthcare systems and organizations are creating larger networks of reciprocal relationships between primary (low-care, i.e., medical domes) and high-care facilities diffused into the cities [72,73,74].

Practical, feasible, repeatable strategies and actions that could be adopted are listed below:

➢ to improve communication with the neighborhoods, in particular regarding the location and times of services, particularly those of primary care;
➢ to make the structures recognizable [75] and pleasing in appearance, both in internal and external spaces;
➢ to provide interactive facilities with the surrounding urban context, in particular regarding the most socially disadvantaged neighborhoods;
➢ to institutionalize the mechanisms for assessing possible spatial or aspatial barriers to access, in an improving perspective, especially in the phases of land and urban planning;
➢ to facilitate the integration between institutions, especially those of proximity, and the local social and associative fabric [76,77].

### 4.10. New Forms of Living, Conscious of Co-Participation Models and Aware of Sharing Quality Objectives

In the Italian context, housing policies have always occupied a marginal part, also taking into account the sheer number of properties nationwide. [78]. However, two negative events, real estate speculation and immediately afterwards, the economic crisis, have been largely responsible for bringing about the housing discomfort that now affects increasingly large sections of the population [79]. This is the framework in which we can see a renewed interest in the theme of living and its new forms, especially in large cities, closely related to a conceptual shift of focus from policies for the home toward policies for living [80]. It is necessary that such policies guarantee citizens not only an access to decorous housing, but also the right to live in a context that is environmentally, socially, and economically sustainable and that meets the specific needs of users.

Practical, feasible, repeatable strategies and actions that could be adopted are listed below:

➢ to define housing standards that guarantee energy efficiency, healthiness, and livability;
➢ to focus on the implementation of effective and lasting social housing interventions through the optimization of the high quality/low cost ratio and high living comfort;
➢ to improve the housing condition, also intervening in the relational dimension of the tenants;
➢ to promote the experimentation of new or renewed forms of living, in which the inhabitants are called to participate actively in the construction of a sustainable community;
➢ to encourage housing projects that, thanks to the presence of particular services (housing assistance for elderly and disabled, babysitting, etc.), are able to accommodate categories of users who are not entirely self-sufficient or need assistance;
➢ to encourage housing projects focused on the direct and active involvement of residents both in the design and construction phase, and in the maintenance phase, such as self-construction and cohousing projects;
➢ to promote education for space-sharing and co-management of services, with a view of socializing and saving money.

## 5. Conclusions

Considering the “Urban Health Rome Declaration” discussed in Rome (December, 2017) at the European meeting “G7 Health”, which defines the strategic aspects and action to improve health in cities through both a holistic and multi-sectorial approach to public health promotion policies within the urban context, the authors have worked to define an updated integration of the strategic goals for achieving a “Healthy and Salutogenic City” useful to designers, local governments and public bodies, policy makers, and all professionals working at local health agencies.

The design strategies and health policies identified underline that the connection between morphological and functional features of urban context and public health is crucial for contemporary cities and modern societies [18]. Urbanization and city shaping provide substantial opportunities for public health promotion and protection [81], but can also become a source of risks that represent a main concern for national health systems, given the large portion of the population potentially involved. For example, the population density that characterizes urban areas is changing public health perspectives, in terms of both issues and possible solutions. Since the Ottawa Charter and the strategies “Health in All Policies”, the environment and living spaces have been considered a global, social, and political entity that strongly influences the population’s health status.

What is the contemporary challenge? Researchers and practitioners, from both technical and medical backgrounds, have identified the need for an interdisciplinary and trans-disciplinary approach, in order to address the main health problems of the city and of contemporary society [82]; joint action is required in order to involve communities, starting from the professionals themselves. We should take account of urban health and sustainability from the early stages of urban planning, given that urban planning can and must serve as primary prevention and collaboration in promoting health, highlighting the need for a holistic approach to the city’s construction. This bottom-up approach is one of the emerging health challenges for all contemporary cities and the related health systems of the nations involved.

## Figures and Tables

**Table 1:** The key points included in the Erice 50 Charter [19].

Strategies for Diseases Prevention and Health Promotion in Urban Areas	
Key points	(1). Promoting urban planning interventions that point citizens towards healthy behaviors
	(2). Improving living conditions in the urban context
	(3). Building an accessible and inclusive city, with a special focus on the elderly population
	(4). Encouraging the foundation of resilient urban areas
	(5). Supporting the development of new economies and employment through urban renewal interventions
	(6). Tackling social inequalities
	(7). Improving stakeholders’ awareness of the factors affecting public health in the cities
	(8). Ensuring a participated urban governance
	(9). Introducing qualitative and quantitative performance tools, capable of measuring the city’s attitude to promoting healthy lifestyles and to monitoring the population’s health status
	(10). Encouraging the sharing of knowledge and accessibility to information

**Table 2:** Objectives and key points for healthy design and urban planning strategies.

Healthy Design and Urban Planning Strategies, Actions, and Policy
Environmental and social sustainability
1. Land consumption, sprawl, and shrinking cities;
2. Urban solid waste management;
3. Energy aspects and environmental planning at an urban scale;
4. Socio-assistance and welfare network at an urban scale: importance of a rational and widespread system.
Adaptation to climatic changes
5. Climate change and management of adverse weather events;
6. Tactical urbanism and urban resilience;
7. Housing emergencies in relation to socio-economic and environmental changes.
New responses to the population’s needs
8. New forms of living, conscious of co-participation models and aware of sharing quality objectives.
9. Urban comfort, safety, and security perception;
10. Strengths and weaknesses of urban green areas and infrastructures.
